# Geophysical and Hydro-Chemical Investigations of Oke Asunle Dumpsite in Ile-Ife, Southwestern Nigeria for Subsoil and Surface Water Pollution

**DOI:** 10.5696/2156-9614-8.20.181209

**Published:** 2018-12-03

**Authors:** Awoibi Joe-Ukairo, Ademakinwa G. Oni

**Affiliations:** 1 Nigeria Hydrological Services Agency, Federal Ministry of Water Resources, Abuja, Nigeria; 2 Department of Geology, Obafemi Awolowo University, Ile-Ife, Osun State, Nigeria

**Keywords:** dumpsite, leachate, geophysical investigation, hydro-chemical analysis, subsoils, surface water, pollution

## Abstract

**Background.:**

Waste deposited in dumpsites immediately becomes part of the hydrological system. Over time, waste components bio-accumulate and/or decompose into contaminant liquid, leading to pollution of soil and water and posing a risk to human health.

**Objectives.:**

The present study employed integrated hydro-chemical and geophysical methods to assess surface water and soil/subsoil within the premises of the Obafemi Awolowo University waste dumpsite in Ile-Ife, southwest Nigeria, for possible leachate pollution.

**Methods.:**

The electrical resistivity method involving 1D vertical electrical sounding (VES) and 2D dipole-dipole profiling techniques and hydro-chemical analysis were used. Two-dimensional profiling data were gathered along two orthogonal traverses and inverted into 2D resistivity images. Schlumberger VES data were gathered and quantitatively interpreted using partial curve matching and computer assisted 1D forward modeling. Hydro-chemical analysis was carried out on three water samples collected from the Asunle River for water quality testing. Anthropogenic pollution determinant parameters such as pH, conductivity, total dissolved solid, cations (calcium, magnesium, sodium, and potassium), anions (chloride, sulphate, biocarbonate, and nitrate) and the less abundant heavy metals in granitic gneiss-derived soil such as cadmium, copper, iron, manganese and lead were analyzed.

**Results.:**

Three geologic layers: topsoil, weathered basement and fresh basement were identified. Within the topsoil and weathered layer, two zones with contrasting geoelectrical characteristics were observed. The first zone, outside the dumpsite boundary, was characterized by relatively high resistivities (78–178 Ωm), typical of unimpacted soil. The second zone, within the dumpsite boundary, was characterized by relatively low resistivity values (15–47 Ωm) up to depth levels between 2.5 and > 15 m. The analyzed physico-chemical parameters, except for turbidity, fell within set limits for potable water quality. However, the concentration levels of heavy metals such as cadmium (0.017 - 0.018 mg/l); iron (0.544 - 0.739 mg/l) and lead (0.501 -0.551 mg/l) significantly exceeded standard limits.

**Conclusions.:**

The results of the present study indicate that subsoil and surface water within and around the dumpsite can be considered to be polluted.

**Competing Interests.:**

The authors declare no competing financial interests.

## Introduction

Natural resources such as water, coal, oil, gas and vegetation are vital to economic development and sustainability.^1^ The extraction, processing and eventual use of these resources can cause environmental problems such as the disruption/or destruction of the ecosystem, decreased biodiversity and pollution of subsoil, water resources and air.^2^ High-income countries produce the most waste per capita, while low income countries produce the least.^3^ The level of technology for waste disposal varies, as developed countries have more organized systems for waste management compared to developing and underdeveloped countries.^4^ Thus, the impact of disposed wastes on the environment is greater in underdeveloped countries.^5^,^6^

Waste deposited in landfills or refuse dump sites immediately becomes part of the hydrological system. While some components of waste are not degradable and bio-accumulate over time, others will decompose/or degrade into liquid contaminants.^7^,^8^,^9^

Leachate can percolate and/or migrate via gravity and runoff through soil/subsoil into the surface and groundwater, thereby polluting the soil/subsoil and water resources. Contamination of surface and groundwater by leachate from open dumpsites, particularly in urban areas, has become an increasing problem for residents in developing countries.^10^ Open dumping is an improper method of waste disposal that threatens the health and safety of local residents as it attract mosquitoes and flies that can carry disease.^11^ In addition to the unpleasant sight and foul odor of open dumps, they also violate waste management laws.

The Obafemi Awolowo University (OAU), Ile-Ife, Osun State, Nigeria is one of the largest universities in Nigeria.^12^ Its unprecedented growth rate has lead to an increase in waste generated on campus.^13^ Hydro-geoenvironmental studies at the university sewage disposal site showed that the groundwater within the study area had been contaminated. The focus of the present study is OAU's main dumpsite, an open waste dumpsite that has been collecting wastes for over fifty years, leading to the generation of leachate plumes and possible impacts on the surrounding and underlying subsoil and water resources (surface and groundwater). The present study employed integrated hydro-chemical and geophysical methods to assess the surface water and soil/subsoil within the Obafemi Awolowo University dumpsite in Ile-Ife, southwest Nigeria, for possible leachate pollution.

## Methods

Integrated geophysical survey and hydro-chemical analysis were used for the present study.

### Study area

**Figure 1:**
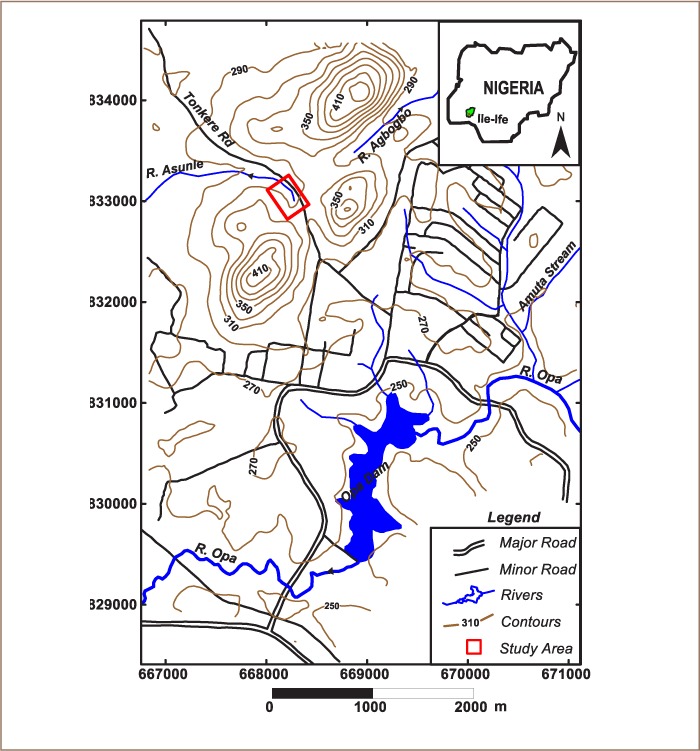
Map of Obafemi Awolowo University campus showing the study area

The Obafemi Awolowo University waste dumpsite (Oke Asunle dumpsite) was established more than fifty years ago. It is located in the northern part of the campus and lies within latitudes 7° 31′ 57″ N to 7o 32′ 11″ N and longitudes 4° 31′ 22″ E to 4° 31′ 32″ E (*Figure 1*). The dumpsite contains mainly domestic wastes, most of which are biodegradable. The topography of the study area is gently undulating with surface elevation that ranges between 310 and 320 m above sea level (*Figure 1*). The study area falls within the tropical climatic zone characterized by a short dry season (November–March) and a long wet season (April–October) with a mean annual rainfall of 1,600 mm.^14^

**Abbreviations**
*FME*: Federal Ministry of Environment
*NIS*: Nigeria Industrial Standard
*OAU*: Obafemi Awolowo University
*TDS*: Total dissolved solids
*TR*: Orthogonal traverses
*VES*: Vertical electrical sounding
*WHO*: World Health Organization

**Figure 2:**
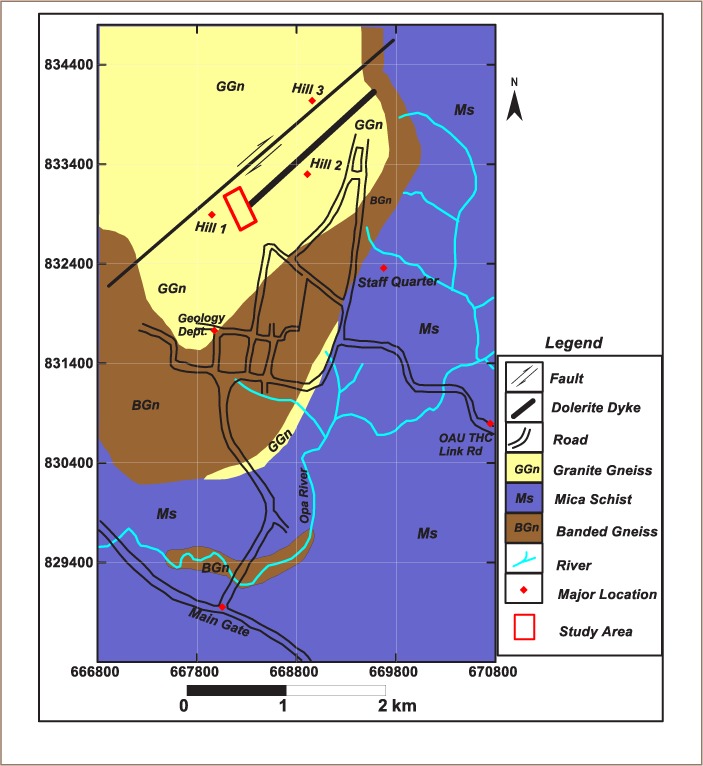
Geological map of Obafemi Awolowo University campus showing the study area (modified from Boesse, 1989)^17^

Average annual temperatures fall between 18°C and 33°C with relatively high humidity.^15^,^16^ The vegetation consists of dense evergreen forest with a variety of hardwood timbers and grasses. Campus effluent drains into the Opas river, the major river within the study area. Other nearby rivers include Agbogbo and Asunle, whose source is the spring behind the dumpsite. The OAU campus is underlain by the Ife-Ilesha schist belt whose major lithological units include granite gneiss, banded gneiss and mica schist (*Figure 2*).^17^ The waste dumpsite is underlain by granite gneiss which is categorized within the migmatite–gneiss–quartzite complex.

### Geophysical and hydro-chemical investigation

**Figure 3:**
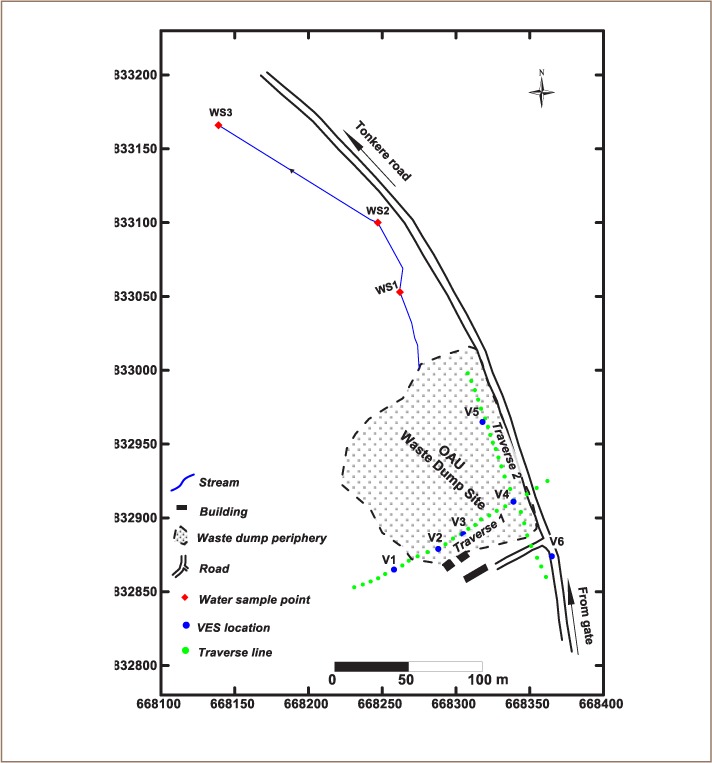
Base/data acquisition map of the study area showing the waste dump boundary, geophysical traverse lines, VES and surface water sample locations Abbreviations: TR, traverse line; V, VES location, WS, water sample point

Two orthogonal traverses (TR 1 and TR 2) of 150 m in length were established, and 1D vertical electrical sounding (VES) and 2D dipole-dipole data were gathered (Figure 3). The 2D dipole-dipole data were acquired at 5 m inter-electrode spacing and inter-dipole separation factor (n) that varied from 1–5. The 2D data were gathered as 2D pseudosections and inverted into 2D resistivity images using DIPRO for windows V. 4.0 software. Six vertical electrical soundings utilizing the Schlumberger array were carried out and presented as VES curves (*Figure 3*). The VES curves were interpreted using partial curve matching and computer assisted 1D forward modeling using the WinResist software. The VES interpretation results (layer resistivities and thicknesses) were used to generate 2D geoelectric sections. Hydro-chemical analysis was performed on three surface water samples collected from River Asunle located within the study area using standard methods (*Figure 3*).^18^ Water quality parameters were analyzed for color, turbidity, pH, conductivity, total dissolved solids (TDS), alkalinity, total hardness, cations and anions and trace metals. The analyzed cations included calcium (Ca^2+^), magnesium (Mg^2+^), sodium (Na+), and potassium (K+) while the anions included chloride (Cl−), sulphate (SO_4_^2−^), biocarbonate (HCO_3−_), and nitrate (NO_3−_). Trace metals such as cadmium (Cd), copper (Cu), iron (Fe), manganese (Mn) and lead (Pb) were also analyzed due to their high concentration in waste materials identified within the dumpsite and their relatively low abundance in granite gneiss rock that underlay the study area.

## Results

**Figure 4 —.**
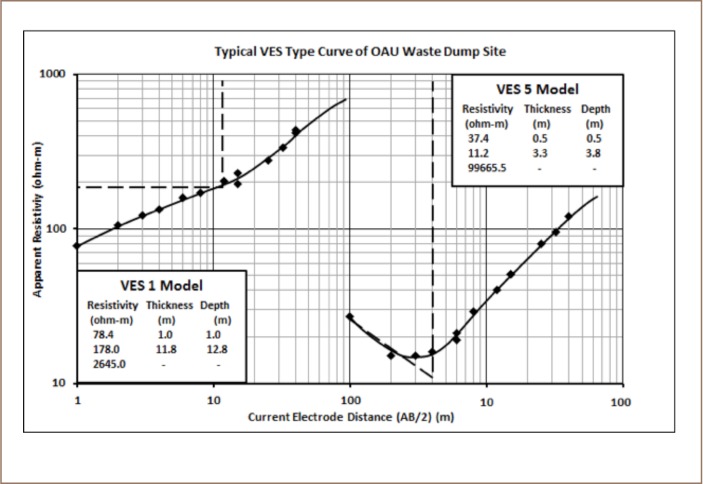
Typical VES type curves within the study area

**Figure 5(a–b):**
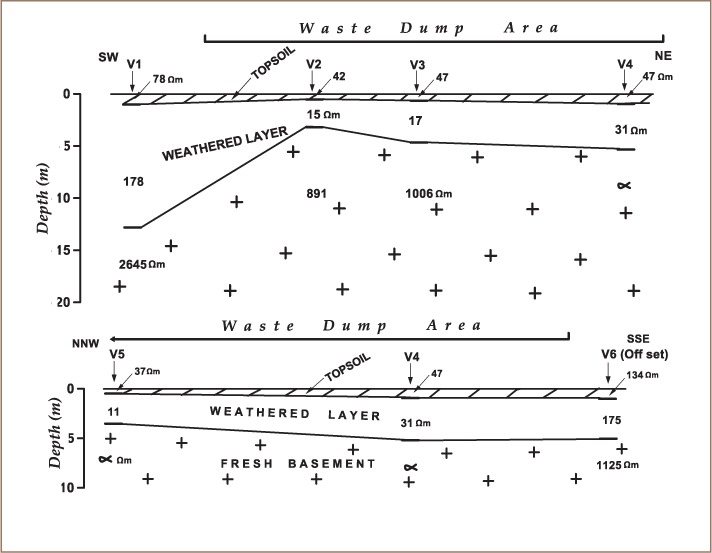
Geoelectric sections along traverses (a) TR 1 and (b) TR 2

**Figure 6 (a–c):**
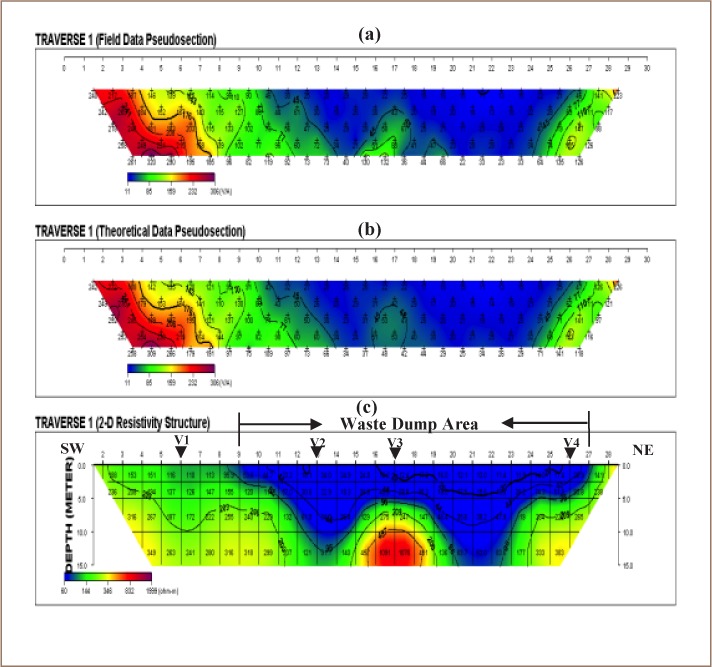
(a) Field observed and (b) theoretical pseudosections and (c) 2D resistivity structure along traverse (TR 1)

**Figure 7 (a–c):**
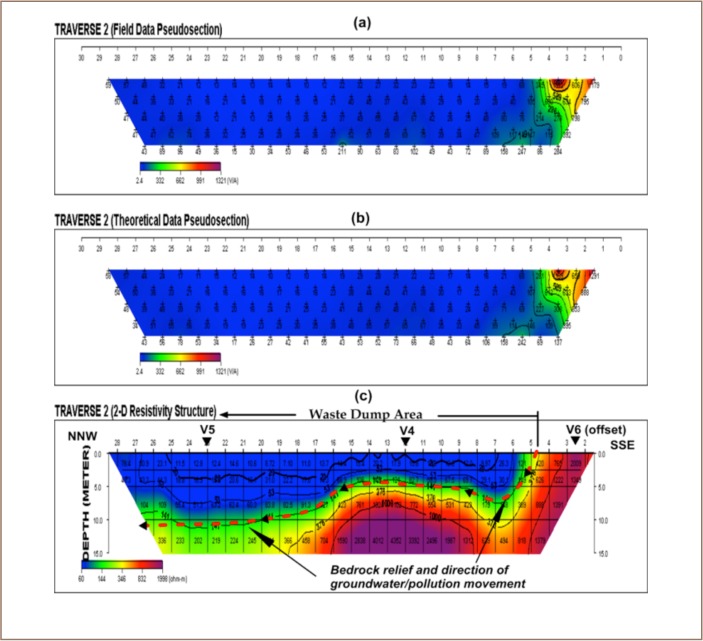
(a) Field observed and (b) theoretical pseudosections and (c) 2D resistivity structure along traverse (TR 2)

The VES model resistivity type curve identified in the study area are presented in Figure 4, while the geoelectric sections generated from VES along traverses 1 and 2 are displayed in Figures 5(a–b). The underlying subsurface sequences were identified from the geoelectric sections. A comparative analysis of the geoelectric characteristics (resistivity values) of layers within and outside the waste dumpsite is shown in Table 1. Figures 6(a–c) and 7(a–c) display the field, theoretical pseudosections and inverted resistivity structures of the 2D dipole-dipole survey along traverses 1 and 2. The images revealed the subsurface layers underlying the study area and the variations in resistivity within and outside the dumpsite boundary.

**Table 1:**
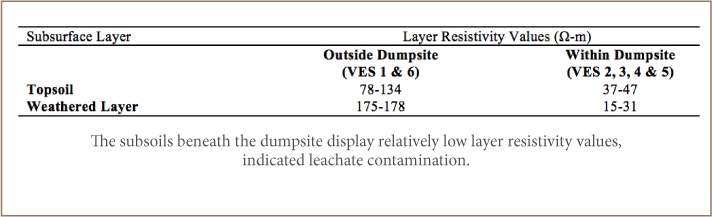
Geoelectric Characteristics of VES Locations Outside and Within the Dumpsite

### Hydro-chemical analysis results

**Figure 8:**
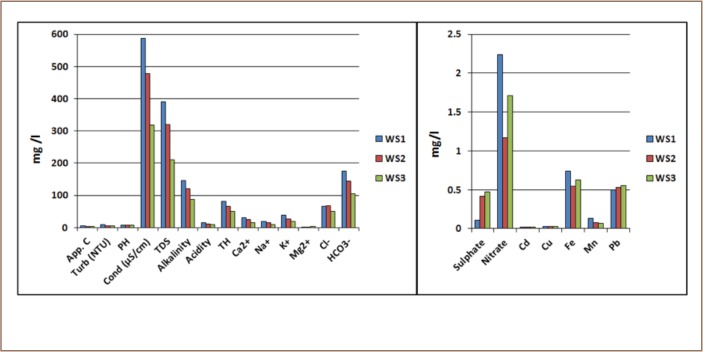
Variation in elemental concentrations of analyzed surface water samples with increasing distance from dumpsite

Table 2 presents the summary of the results of the hydro-chemical analysis of water samples. Figure 8 shows the relationship in the elemental concentration of the analyzed parameters in proximity to the dumpsite, with water sample 1 being the closest and water sample 3 the furthest from the dumpsite.

**Table 2:**
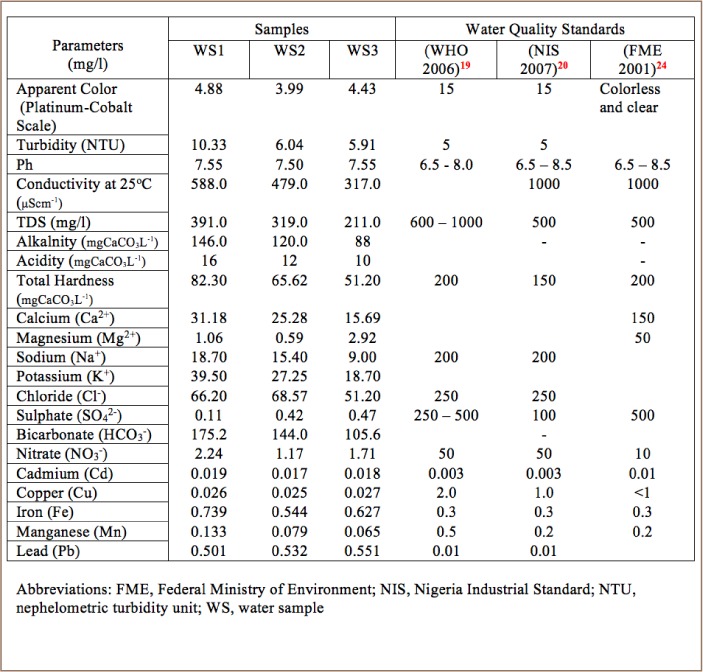
Hydro-Chemical Analysis Results of Surface Water Samples

## Discussion

Two VES curve types, H and A types, with 3 geoelectric layers, were identified within and outside the waste dumpsite (*Figure 4*). The geoelectric section beneath TR 1 (*Figure 5a*) identified 3 geologic layers. These layers include topsoil, weathered layer and fresh basement. The topsoil resistivity values varied between 42 and 78 Ω-m with a thickness between 0.6 and 1.0 m. The topsoil layer was composed of clay. The underlying weathered layer was made up of a mixture of clay and sand with resistivity between 15 and 178 Ω-m and thickness between 2.6 and 11.8 m. The fresh basement had resistivity values from 891 Ω-m to infinity. The depth of the bedrock/basement layer varied between 3.2 and 12.8 m. The weathered layer constitutes the aquifer. The topsoil and weathered layer resistivity values beneath VES 1, located outside the dumpsite (78 and 178 Ω-m, respectively) were relatively higher than those within the dumpsite, VES 2, 3 and 4, (42–47 Ω-m for topsoil and 15–31 Ω-m for weathered layer). Vertical electrical sounding 1 also displayed a different curve type (A-type) and increasing layer resistivity with depth compared with VES 5 within the waste dumpsite which showed a typical H-type curve with decreased layer resistivity at shallow depths. Along TR 2, the section revealed three geologic layers, comprised of a clay/sandy clay topsoil with resistivities and thicknesses that varied from 37–134 Ω-m and 0.5–1.0 m, respectively; a clay and sandy clay weathered layer with resistivity and thickness values of 11–175 Ω-m and 3.3–4.3 m, respectively; and a fresh basement with resistivity values between 1125 Ω-m to infinity (*Figure 5b*). The topsoil and weathered layers (overburden) thickness ranged from 3.8 to 5.3 m. In addition, VES 6 located outside the dumpsite is characterized by an A-type curve with higher resistivity values in topsoil and weathered layers (134 and 175 Ω-m, respectively) compared to VES 4 and 5, which were characterized with an H-type curve within the dumpsite (37–47 Ω-m for topsoil and 11–31 Ω-m for weathered layer). Table 1 compares the geoelectrical characteristics within and outside of the dumpsite.

### Two-dimensional resistivity images

The 2D resistivity structure along TR 1 revealed that outside the dumpsite, the traverse is underlain by two geologic units (*Figure 6c*). These are the topsoil/weathered layers (blue/green color band) and fresh basement (yellow/purple color band). The topsoil and weathered layer merge to constitute the overburden with thicknesses of up to 15 m. The topsoil/weathered layer resistivity values (> 110 Ω-m) outside the dumpsite are higher than those within the waste dumpsite with resistivities generally less than 54 Ω-m. The low resistivity zones (blue color band) between stations 9 and 27 correlated perfectly with the field observed boundary of the dumpsite and hence could be identified as the source of polluted leachate between 2.5 and > 15 m. The results also indicate a vertical migration of leachate plume with little or no lateral migration. Near-surface high resistivity fresh basement rock (yellow/red/purple color bands) is observed to underlay stations 1–5, outside the waste dump, from the 2D resistivity structure along Traverse 2 (*Figure 7c*). This result is supported by the existence of a low-lying outcrop between these stations with limited superficial deposit. However, within the dumpsite (stations 5 – 28), the depth to the basement bedrock varies between 4 m and 10 m. The overburden materials (topsoil and weathered layer) could not be differentiated from the 2D image as these layers seem to have merged due to overlapping low resistivity arising from contaminant plume emanating from the waste.^18^ The low resistivity zone (blue color band) with values generally less than 91 Ω-m and thicknesses ranging from 3 m to 10 m indicates the impacted zone which falls within the dumpsite boundary. Although Figure 7(c) shows that migration of the pollution plume is essentially vertical, there is evidence of significant lateral migration outside the waste dumpsite boundary, towards the north-northwest direction, suspected to be the direction in which groundwater flows.

In a basement complex environment, groundwater flow is determined by the gradient of basement bedrock. Groundwater flows from zones of high to low bedrock relief. Figure 7(c) shows that the basement bedrock boundary (in greenish/yellowish/red color) dips north-northwestward.

### Hydro-chemical analysis results

Detailed discussion of the results of the hydro-chemical analysis (*Table 2*) from sampled surface water is presented as follows:

### Color and turbidity

The color rating of the analyzed samples using the platinum-cobalt scale ranged from 3.99 and 4.88, while turbidity varied from 5.91 to 10.33 nephelometric turbidity units. The color rating fell within the World Health Organization (WHO) and the Nigeria Industrial Standard (NIS) acceptable limit of 15 and with the exception of water sample 3, showed a decreasing trend as the distance from the waste dumpsite increased (Figure 8).^19^,^20^ For turbidity, all the samples showed higher values (5.91 – 10.33 nephelometric turbidity unit) than the standard threshold of 5 (*Table 2*). However, turbidity decreased with increased distance from the dumpsite (*Figure 8*).

### pH and conductivity

The pH values of the water samples varied from 7.50 to 7.55 and hence were slightly basic. No definable trend was observed in the pH values (*Figure 8*). Water samples 1 and 3 had the highest pH value of 7.55, while water sample 2 had the lowest pH value of 7.50. However, the pH values fell within the permissible range of 6.5–8.5 for potable water. The conductivity values generally decreased as the distance increased from the dumpsite (*Figure 8*). The conductivity values ranged from 317 to 588 μScm^−1^ and all fell within the permissible level.

Other physical parameters such as TDS, alkalinity, acidity and total hardness showed a similar trend of decrease in their concentration levels as distance increased from the dumpsite (*Figure 8*). The concentration levels recorded for TDS, alkalinity, acidity and total hardness were 211 – 391 mg/l; 88 – 146 mgCaCO_3_/l; 10 – 16 mgCaCO_3_/l and 51.2 – 82.3 mgCaCO_3_/l, respectively. The decreasing trend of the anthropogenic pollution determinant parameters such as conductivity and TDS implies a point source of pollution, suggesting the dumpsite as the pollution source.^21–23^

### Cations

The results of the analyzed cations showed that the concentration levels of Ca^2+^, Mg^2+^, Na+, and K+ in the water samples varied from 15.69 – 31.18 mg/l; 0.59 – 2.92 mg/l; 9.0 – 18.7 mg/l and 18.7 – 39.5 mg/l, respectively. The general decrease in the values of each cation, with the exception of Mg^2+^, as the distance increased from the dumpsite suggests that the source of these ions are from contaminant leachate emanating from the dumpsite (*Figure 8*). Magnesium is mainly derived from rocks/soils and this may explain the variable trend displayed by the cation. Analyzed cation concentration levels all fell within the Federal Ministry of Environment (FME), WHO and NIS limits for potable water.^19^,^20^,^24^

### Anions

Concentration levels of 51.2 – 68.57 mg/l; 0.11 – 0.47 mg/l, 105.6 – 175.2 mg/l and 1.17 – 2.24 mg/l for Cl, SO_4_^2−^, HCO_3−_ and NO_3−_, respectively, mean that analyzed water samples are safe for consumption as these values met the standard thresholds for drinking water. However, the general trend in which sites closest to the dumpsite showed the highest concentrations of ions and decreased with increasing distance from the dumpsite indicates that the dumpsite is a point source of pollution (*Figure 8*).

### Heavy Metals

Heavy metals concentration levels were 0.017 -0.018 mg/l for Cd; 0.025 – 0.027 mg/l for Cu; 0.544 - 0.739 mg/l for Fe; 0.065 – 0.133 mg/l for Mn, and 0.501 – 0.551 mg/l for Pb. Heavy metal concentration levels were observed to be significantly higher than the FME, WHO and NIS permissible levels for potable water, except for Cu and Mn.^19^,^20^,^24^

Although the results of the hydro-chemical analysis showed that the physical and chemical parameters, with the exception of turbidity, fell within the FME, WHO and NIS thresholds for potable water, the concentration levels of heavy metals such as Cd, Fe and Pb significantly exceeded their limits.^19^,^20^,^24^ This indicates that surface water is highly polluted with heavy metals.

## Conclusions

Geophysical and hydro-chemical investigations of the Obafemi Awolowo University, Ile-Ife, dumpsite were carried out to assess possible pollution of subsoil and surface water within the study area. The results of the geophysical investigation identified three geologic layers: topsoil, weathered layer and fresh basement. Within the topsoil and weathered layer (overburden), two zones with distinct geoelectrical characteristics were identified. The first zone (outside the dumpsite boundary) was characterized by an A-type curve with increasing layer resistivities with depth, typical of unimpacted soil, and relatively high resistivity, 78/178 Ω-m, in the topsoil/weathered layer. The second zone (within the dumpsite boundary) was characterized by an H-type curve with decreasing layer resistivities at shallow depth and relatively low resistivity, 47/15 Ω-m, in the topsoil/weathered layer, characteristic of impacted soil at depths ranging between 2.5 and > 15 m. Hydro-chemical analysis results of sampled surface water revealed a decreasing trend of elemental concentrations of analyzed parameters as distance from the dumpsite increased. Although the analyzed physical and chemical parameters, with the exception of turbidity, fell within the permissible levels for potable water, the concentration levels of heavy metals such as Cd, Fe and Pb significantly exceeded acceptable limits. The results of the present study therefore indicate that subsoil and surface water within and around the waste dumpsite are polluted.

## References

[1] Everett T, Ishwaran M, Ansaloni GP, Rubin A (2010). Economic growth and the environment.

[2] Gutti B, Aji MM, Magaji G (2012). Environmental impact of natural resources exploitation in Nigeria and the way forward. J Appl Technol Environ Sanit.

[3] Daskalopoulous E, Badr O, Probert SD (1998). An integrated approach to municipal solid waste management. Resour Conserv Recycl [Internet].

[4] Eggleston S, Buendia L, Miwa K, Ngara T, Tanabe K (2006). 2006 IPCC guidelines for national greenhouse gas inventories. Vol. 5, Waste [Internet].

[5] Nordin AP, da Silva J, de Souza CT, Niekraszewicz LAB, Dias JF, da Boit K, Oliveira MLS, Grivicich I, Garcia ALH, Oliveira LFS, da Silva FR (2018). In vitro genotoxic effect of secondary minerals crystallized in rocks from coal mine drainage. J Hazard Mater [Internet].

[6] Ramos CG, Querol X, Dalmora AC, de Jesus Pires KC, Schneider IA, Oliveira LF, Kautzmann RM (2017). Evaluation of the potential of volcanic rock waste from southern Brazil as a natural soil fertilizer. J Clean Prod [Internet].

[7] Gredilla A, de Vallejuelo SF, Gomez-Nubla L, Carrero JA, de Leao FB, Madariaga JM, Silva LF (2017). Are children playgrounds safe play areas? Inorganic analysis and lead isotope ratios for contamination assessment in recreational (Brazilian) parks. Environ Sci Pollut Res [Internet].

[8] Dutta M, Saikia J, Taffarel SR, Waanders FB, de Medeiros D, Cutruneo CM, Silva LF, Saikia BK (2017). Environmental assessment and nano-mineralogical characterization of coal, overburden and sediment from Indian coal mining acid drainage. Geosci Front [Internet].

[9] de Vallejuelo SF, Gredilla A, da Boit K, Teixeira EC, Sampaio CH, Madariaga JM, Silva LF (2017). Nanominerals and potentially hazardous elements from coal cleaning rejects of abandoned mines: Environmental impact and risk assessment. Chemosphere [Internet].

[10] Atlas RM (1988). Microbiology: fundamentals and applications.

[11] Ekeocha NE, Ikoro DO, Okonkwo SE (2012). Electrical resistivity investigation of solid waste dumpsite at Rumuekpolu in Obio Akpor L.G.A., River State, Nigeria. Int J Sci Technol.

[12] (2012). Committee on needs assessment of Nigerian public universities.

[13] Adepelumi A, Ako B, Ajayi T (2001). Groundwater contamination in the basement-complex area of Ile-Ife, southwestern Nigeria: a case study using the electrical resistivity geophysical method. Hydrogeol J [Internet].

[14] Iloeje NP (1981). A new geography of Nigeria.

[15] Adeleke BO, Goh CL (1978). Certificate of physical and human geography.

[16] (2007). Daily weather forecast on the Nigerian Television Authority.

[17] Boesse JM A geologic map of the Obafemi Awolowo University Campus [thesis].

[18] Odipe OE, Ogunleye RA, Sulaiman M, Abubakar SS, Olorunfemi MO (2018). Integrated geophysical and hydro-chemical investigation of impact of the Ijemikin waste dumpsite in Akure, southwestern Nigeria, on groundwater quality. J Health Pollut [Internet].

[19] (1996). Guidelines for drinking water quality. Health criteria and other supporting information [Internet].

[20] (2007). Nigeria standard for drinking water quality.

[21] Cutruneo MN, Oliveira ML, Ward CR, Hower JC, de Brum IA, Sampaio CH, Kautzmann RM, Taffarel SR, Teixeira EC, Silva LF (2014). A mineralogical and geochemical study of three Brazilian coal cleaning rejects: demonstration of electron beam applications. Int J Coal Geol [Internet].

[22] Dias CL, Oliveira ML, Hower JC, Taffarel SR, Kautzmann RM, Silva LF (2014). Nanominerals and ultrafine particles from coal fires from Santa Catarina, South Brazil. Int J Coal Geol [Internet].

[23] Kronbauer MA, Izquierdo M, Dai S, Waanders FB, Wagner NJ, Mastalerz M, Hower JC, Oliveira ML, Taffarel SR, Bizani D, Silva LF (2013). Geochemistry of ultra-fine and nano-compounds in coal gasification ashes: a synoptic view. Sci Total Environ [Internet].

[24] (2001). Guidelines and standard for water quality in Nigeria publication of Federal Ministry of Environment.

